# Admission Serum Magnesium Levels Is Associated with Short and Long-Term Clinical Outcomes in COVID-19 Patients

**DOI:** 10.3390/nu15092016

**Published:** 2023-04-22

**Authors:** Amitai Segev, Adam Sagir, Shlomi Matetzky, Amit Segev, Shaul Atar, Michael Shechter

**Affiliations:** 1 The Leviev Cardiothoracic & Vascular Center, Chaim Sheba Medical Center, Ramat Gan 5236723, Israel; shlomi.matetzky@sheba.health.gov.il (S.M.); amit.segev@sheba.health.gov.il (A.S.); shechtes@netvision.net.il (M.S.); 2 The Sackler Faculty of Medicine, Tel Aviv University, Tel Aviv 6997801, Israel; 3 Cardiovascular Division, Galilee Medical Center, Nahariya 2210001, Israel; adam.sagir@gmail.com (A.S.); shaula@gmc.gov.il (S.A.); 4 Azrieli Faculty of Medicine, Bar Ilan University, Ramat Gan 5290002, Israel

**Keywords:** COVID-19, magnesium, endothelial dysfunction, prognosis, mortality

## Abstract

Background: In the face of the global pandemic that the coronavirus disease 2019 (COVID-19) has created, readily available prognostic markers may be of great use. Objective: To evaluate the association between serum magnesium (sMg) levels on admission and clinical outcomes in hospitalized COVID-19 patients. Methods: We retrospectively analyzed all patients admitted to a single tertiary center with a primary de novo diagnosis of COVID-19. Patients were followed for a mean of 10 ± 7 months. Demographic, clinical and laboratory data were collected and compared between five groups of patients according to sMg quintiles on hospital admission. Results: The cohort included 1522 patients (58% male, 69 ± 17 years old). A low sMg level (1st quintile) was associated with higher rates of diabetes and steroid use, whereas a high sMg level (5th quintile) was associated with dyslipidemia, renal dysfunction, higher levels of inflammatory markers and stay in the intensive care unit. All-cause in-hospital and long-term mortality was higher in patients with both low and high sMg levels, compared with mid-range sMg levels (2nd, 3rd and 4th quintiles; 19% and 30% vs. 9.5%, 10.7% and 17.8% and 35% and 45.3% vs. 23%, 26.8% and 27.3% respectively; *p* < 0.001 for all). After adjusting for significant clinical parameters indicating severe disease and renal dysfunction, only low sMg state was independently associated with increased mortality (HR = 1.57, *p* < 0.001). Conclusions: Both low and high sMg levels were associated with increased mortality in a large cohort of hospitalized COVID-19 patients. However, after correction for renal dysfunction and disease severity, only low sMg maintained its prognostic ability.

## 1. Introduction

The coronavirus disease 2019 (COVID-19) has created a global pandemic, with millions infected and about 6.8 million deaths globally [1]. However, there is still a need for readily available markers of disease severity. Since the outbreak, it has become evident that several risk factors, including age, hypertension, diabetes and coronary artery disease, predispose to severe disease with increased mortality [2,3]. These particular risk factors are also associated with a low magnesium state [4,5,6], resulting in an apparent overlap between risk factors for severe COVID-19 and states associated with hypomagnesemia.

Serum magnesium makes up only about 1% of the total magnesium stores in our bodies. In the absence of a more selective, reliable and easily testable biomarker, serum magnesium is still the most frequently used laboratory test for evaluating clinical magnesium status [7,8]. A low serum magnesium status reflects total body magnesium depletion, whereas normal serum levels do not necessarily reflect adequate total magnesium. The most common cause of chronic hypomagnesemia is decreased dietary intake, as many processed and fast food contain low magnesium content [8,9]. Moreover, a recent publication reveals significant variability in the normal range of serum magnesium levels around the world [7].

There are several possible mechanisms through which low magnesium levels may worsen the course and outcome during SARS-CoV-2 infection, including endothelial cell damage, alterations in the immune system, thrombus formation and bronchial smooth muscle contraction. Due to the presence of angiotensin converting enzyme 2 receptors, endothelial cells are susceptible to infection by SARS-CoV-2 [10,11], and therefore COVID-19 was postulated to be a systemic disease primarily injuring the vascular endothelium, causing a unique lung injury [12] and a host of thrombotic events [13,14]. It is well established that magnesium plays a major role in maintaining normal endothelial cell function [15] and that magnesium supplementation results in significant improvement in endothelial function [16,17]. Low magnesium levels also predispose to a pro-inflammatory state, with upregulation of inflammatory cytokines, such as interleukin-6 (IL-6) [18] and substance P [19], and decreased activation of vitamin D [20], leading to increased clinical inflammatory response and elevated acute phase reactants, such as C-reactive protein (CRP) and ferritin [21]. In COVID-19 patients, low magnesium was associated with exaggerated immune responses, including ARDS and cytokine storm, both linked to poor prognosis [22]. Moreover, magnesium has been implicated in promoting the formation of atherosclerotic plaques and activation of the coagulation cascade, generating micro and macro vascular thrombi [23]. Indeed, abnormal coagulation parameters, such as increased D-dimer levels, are associated with poor prognosis in COVID-19 patients [14]. Finally, as magnesium has a physiological role as a calcium channel antagonist, magnesium depletion may also cause bronchial spasm [24], exacerbating ventilation-perfusion mismatch and worsen outcomes in COVID-19 patients.

With these considerations in mind, it seems there is a firm pathophysiologic basis linking low magnesium with COVID-19 severity and prognosis. However, clinical data are scarce and includes only a few small observational studies. A retrospective study of 83 COVID-19 patients from Wuhan [25] demonstrated a lower serum magnesium (sMg) level in non-survivors and critically ill patients, compared with those with moderate or severe disease. Similarly, in a prospective study, Quilliot et al. [26] demonstrated a high prevalence of hypomagnesemia in 300 COVID-19 patients, approaching 50%, most notably in critically ill patients, and found an association between hypomagnesemia and infection severity, oxygen therapy and admission to critical care units. Guerrero-Romero et al. (2022) [27] measured the serum magnesium to calcium (Mg-to-Ca) ratio in severe COVID-19 patients. They found that a Mg-to-Ca ratio lower than 0.2 was strongly associated with mortality. In a cohort observational study of 43 COVID-19 hospitalized patients [28], the addition of combined magnesium, vitamin D and vitamin B12 supplementation upon admission was associated with less oxygen therapy.

Our study aims to improve our understanding of sMg’s role in COVID-19 progression and outcomes, by evaluating the association between sMg levels on admission and important clinical outcomes in hospitalized COVID-19 patients. Correlating magnesium levels with clinical sequelae and disease biomarkers can potentially point to a better understanding of risk stratification and management.

## 2. Materials and Methods

We conducted a retrospective cohort study of all patients (≥18 years) with de-novo (primary) diagnosis of COVID-19 and available sMg levels within three days (<72 h) of admission, hospitalized at the Sheba Medical Center between February 2020 and February 2022. Neither patients nor the public were involved in the design, conduct, reporting or dissemination plans of our research. Excluded from the study were patients with diagnosis of solid malignancy, hematologic malignancy and mal-absorption disorders. Data acquisition was approved by the Sheba Medical Center Institutional Review Board (approval number 8554-21-SMC), and informed consent was waived. Following approval, we collected relevant medical history and background diagnoses from the coded electronic medical record. Mortality data were extracted from the Israeli National Population Registry and were available for all cases. Laboratory data were taken from the first available laboratory tests within the index hospitalization. Serum magnesium levels were measured using a photometric color test and are expressed as mg/dl (conversion factor from mg/dL to mmol/L, multiply by 0.4114). Chronic kidney disease (CKD) was defined as an estimated creatinine clearance of less than 60 mL/min/1.73 m^2^, calculated by the CKD-EPI formula upon admission, or by the presence of an appropriate background diagnosis. Patients were divided into five groups according to sMg quintiles upon admission, and their clinical and laboratory parameters were compared.

### Statistical Analysis

All measured variables and derived parameters were summarized by descriptive statistics. For categorical variables, frequency and percentages were presented. For continuous variables, mean and standard deviation (SD) were presented. Subjects were divided into quintiles according to their sMg level upon admission (Q1–Q5) to better clarify non-linear trends. The inequality between group sizes stems from the fact that a high frequency value cannot be split into two quintiles. Comparative testing was performed by one-way ANOVA (or Kolmogorov–Smirnov nonparametric test) for continuous variables and by Chi-square tests for categorical variables. Multivariate analysis of all-cause mortality was performed by Cox regression models, with forward LR method, and presented by the hazard ratios with 95% confidence intervals. The analyses included independent variables/covariates that were statistically significant in the univariate analyses. Un-adjusted and adjusted survival curves are presented. The data were analyzed using SPSS version 28.01. All tests were two-tailed with a significant value of less than 0.05.

## 3. Results

**Figure 1:**
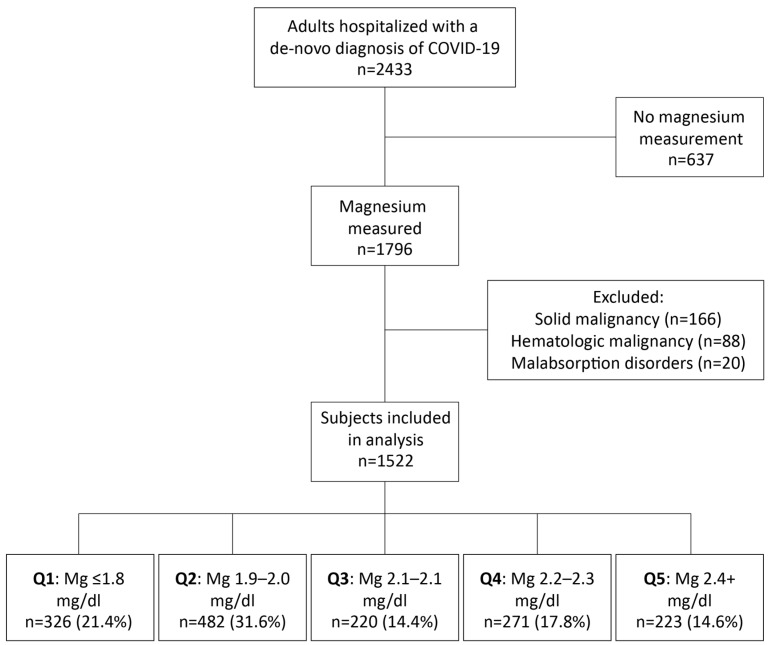
Patient’s flow.

A total of 2433 patients were hospitalized at our medical center with a primary diagnosis of de-novo COVID-19. After excluding patients with malabsorption disorders (*n* = 20), solid malignancy (*n* = 166), hematologic malignancy (*n* = 88) and patients without available sMG level upon admission (*n* = 637), the study cohort consisted of 1522 patients (58% male) with a mean age of 69 ± 17. Our cohort was divided into five groups based on the quintiles of serum magnesium level on admission (Q1–Q5; Figure 1). Patients were followed for a mean of 10 ± 7 months.

### 3.1. Patient Characteristics

Patients baseline characteristics are presented in Table 1. The low sMg group (1^st^ quintile—Q1, *n* = 326) were less likely to be male (50.9% in Q1 vs. 52.7%, 60.5%, 66.8% and 68.6% in Q2–Q5, respectively; *p* < 0.001) and had higher rates of diabetes (23.9% in Q1 vs. 16.4%, 12.7%, 11.8% and 17.5% in Q2–Q5, respectively; *p* < 0.001) and use of proton pump inhibitors (PPIs; 28.2% in Q1 vs. 22.6%, 23.6%, 17.7% and 22% in Q2–Q5, respectively; *p* = 0.049), calcium channel blockers (CCB’s; 24.2% in Q1 vs. 18.3%, 17.7%, 16.6% and 24.2% in Q2–Q5, respectively; *p* = 0.046) and steroids (7.1% in Q1 vs. 4.6%, 2.3%, 3.7% and 1.8% in Q2–Q5, respectively; *p* = 0.016). Patients with high sMg level (Q5, *n* = 223) had higher incidence of CKD at baseline (9.4% in Q5 vs. 6.1%, 3.9%, 5% and 3.3% in Q1–Q4, respectively; *p* = 0.017). Both the low and high sMg level groups (Q1 + Q5) were older compared with the other quintiles (mean of 69 in Q1 and 70 in Q5, compared with 67, 68 and 66 in Q2–Q4, respectively; *p* = 0.039). There was no statistically significant difference between groups with regard to body mass index, as well as underlying comorbidities, including congestive heart failure, hypertension, ischemic heart disease or a history of stroke or transient ischemic attack.

Patients in the fifth quintile (Q5) had significantly higher levels of white blood cells, platelets, sodium, potassium, hepatic enzymes, CRP, ferritin, Lactate Dehydrogenase (LDH) and D-dimer levels, as well as increased creatinine and prolonged international normalized ratio (INR) (*p* < 0.001 for all, Table 1). There was no significant difference in measured troponin levels between groups.

### 3.2. Outcomes

**Figure 2:**
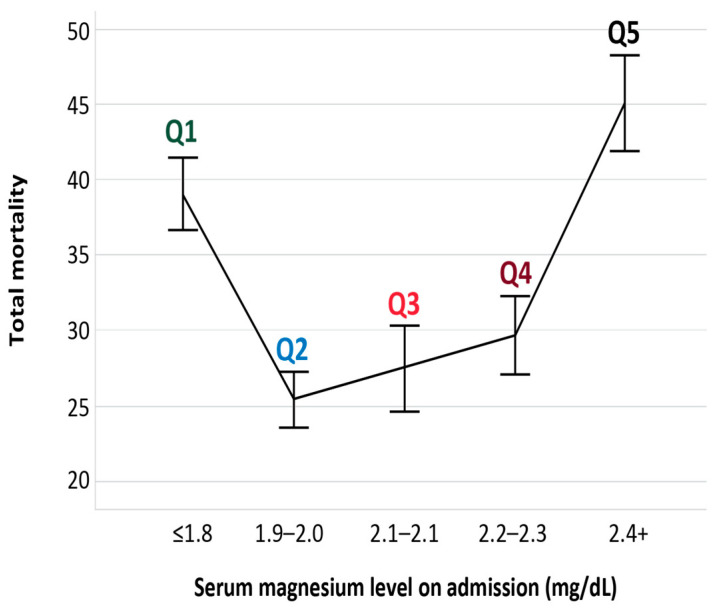
Total mortality by serum magnesium quintiles.

**Figure 3:**
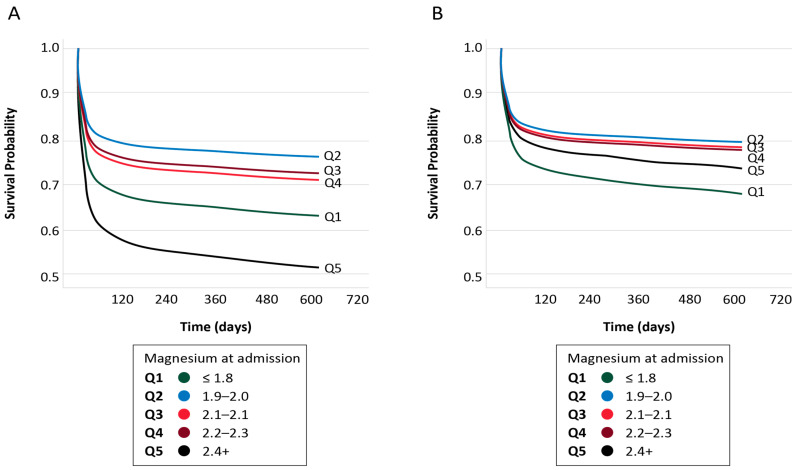
(**A**): Survival curve according to serum magnesium quintiles. (**B**): Survival curve according to serum magnesium quintiles, after multivariate regression model.

The high sMg group (Q5) was associated with higher rates of intensive care unit (ICU) admissions (15% in Q5 vs. 8.9%, 8.1%, 10% and 11.1% and in Q1–Q4, respectively; *p* = 0.007) and with arterial O2 desaturation under 93% (SpO2 ≤ 93%), compatible with the definition of severe COVID according to the World Health Organization (WHO) guidelines [29] (92% in Q5 vs. 84%, 80%, 80% and 85% in Q1–Q4, respectively; *p* < 0.001). Nevertheless, there was no significant difference in length of hospital stay between groups (mean of 10.4 days, 10.6 days, 12.9 days, 10.2 days and 13.4 days in Q1–Q5, respectively; *p* = 0.274). Both low and high sMg levels (Q1 and Q5) were associated with increased total mortality (35.0% and 45.3%, respectively, vs. 23%, 26.8% and 27.3% in Q2, Q3 and Q4, *p* < 0.001) and in-hospital mortality (19% and 30%, respectively, vs. 9.5%, 10.7% and 17.8% in in Q2, Q3 and Q4, *p* < 0.001) (Table 2. See total mortality rates according to quintiles in Figure 2 and survival curves in Figure 3A).

### 3.3. Multivariate Analysis

A multivariate regression model including several clinical and laboratory values, statistically significant in the univariate analysis, was used to compare clinical outcomes between groups. After adjusting for age, diabetes, SpO2 ≤ 93%, creatinine, alanine aminotransferase (ALT), alkaline phosphatase (ALK-P), CRP, LDH and sodium, the increased mortality in the high sMg group (Q5) was not statistically significant, whereas the low magnesium group (Q1) maintained statistically higher mortality rates (when compared to Q2, the quintile with the lowest mortality rate: HR of 1.563 for Q1, 1.102 for Q3, 1.051 for Q4 and 1.288 for Q5) (Table 3. See Survival curves according to sMg quintiles, after multivariate regression model in Figure 3B).

## 4. Discussion

In this study, a U-shaped relationship has been shown between admission sMg levels and mortality in de-novo COVID-19 patients admitted to the hospital, as both low and high admission sMg levels were associated with increased in-hospital and long-term mortality. However, in a multivariate regression model, after adjusting for important clinical factors, only patients with low magnesium had statistically significant increased mortality. Despite the study’s sound pathophysiological basis, there are limited data on the role of magnesium levels in COVID-19 patients. To the best of our knowledge, this is the largest trial undertaken to investigate the role of magnesium in COVID-19 patients.

Currently, there is no international consensus regarding the normal serum magnesium range [7]. Recently, an alternative cutoff for low magnesium was proposed [30,31], whereas a threshold for hypermagnesemia has not yet been established [7]. We therefore divided our cohort into quintiles of magnesium levels, rather than setting a particular threshold for either low or high magnesium. This way, the low and high magnesium groups in our cohort represent a relative finding and not an absolute one.

In our cohort, patients with low sMg had a higher incidence of diabetes, as previously described in non-COVID populations, possibly secondary to alterations in cellular glucose transport, insulin secretion or changes in insulin receptor binding [32]. The low magnesium group also had higher rates of PPI use, a known risk factor for developing hypomagnesemia, likely related to an inhibition of intestinal absorption [33]. Uneven gender distribution across quintiles showed the high magnesium group to have the highest proportion of males. In COVID-19 patients, mortality is indeed higher in men [34] and possibly amongst patients with high magnesium levels [26,35,36].

Mortality rates in our study demonstrated a U-shaped relationship, and in univariate analysis, were significantly higher in both the high and low magnesium groups, compared with other quintiles. However, after multivariate analysis, only the low sMg group maintained its high mortality rates. As previously mentioned, (see Introduction), relying on a plausible pathophysiological basis, a few small observational studies support the association between low sMg levels and poor outcomes in COVID-19 patients [25,26,27,28]. Regarding high sMg levels, a previous study demonstrated that COVID-19 patients with hypermagnesemia had twice the mortality rate, compared to patients with normal magnesium levels [35]. The authors concluded that in light of these results, care should be taken when considering magnesium supplementation in COVID-19 patients. However, it remains unclear whether high magnesium level is a direct causal factor in the progression of the disease or serves as a marker for other poor prognostic factors.

In our study, patients in the high sMg group had higher rates of CKD at baseline and significantly worse renal function upon admission, which are both known to be major risk factors for mortality in COVID-19 [37]. The high sMg levels may be explained by impaired magnesium clearance as typically seen in patients with severe renal dysfunction [38]. Laecke et al. [39] showed that CKD patients with low, rather than high, sMg had a 61% increased mortality risk compared with patients with higher sMg patients (adjusted hazard ratio 1.613; 95% CI, 1.113–2.338; *p* = 0.012). Another trial by Sakaguchi et al. [40] demonstrated a U-shaped relationship between cardiovascular mortality and dysmagnesemia in 142,555 hemodialysis patients. However, while low sMg levels were an independent risk factor for mortality (Odds Ratio = 1.28, CI 95% 1.17, 1.41; *p* = 0.001), after excluding patients with low PTH, possibly secondary to high sMg levels, the mortality in the high sMg group no longer remained significantly higher.

In our cohort, patients in the high sMg group also exhibited elevated markers of inflammation, such as CRP and ferritin, known as poor prognostic factors and associated with severe COVID-19 disease [41]. In addition, the high sMg group also had elevated markers of cell turnover, including potassium and LDH, possibly attributing the high sMg levels in this group, besides renal dysfunction, to rapid mobilization of magnesium from soft tissues in the context of widespread systemic response and sepsis, correlating to infection severity [41,42]. Necrosis due to microvascular thrombosis might be another source of cellular turnover and resulting hypermagnesemia. As COVID-19 is associated with an increase in thromboembolic events [14,23], we indeed witnessed a rise in D-dimer levels in all magnesium groups, with the largest increase in the hypermagnesemia group. Along with a prolonged INR time, two established poor prognostic markers [43], the high sMg group exhibited yet more evidence for severe disease and sepsis induced coagulopathy [44]. The higher sodium levels in the high sMg group may also allude to the severity of infection in this group, as it was previously shown to be associated with sepsis on admission [45]. Indeed, beyond the forementioned laboratory derangements, our data showed that high sMg was associated with a more severe disease state reflected by higher rates of oxygen desaturation and stay in the intensive care unit. Importantly, this connection was not evident with the low sMg group. These findings led us to hypothesize that the measured magnesium level in the hypermagnesemia group was due to both renal dysfunction and a shift between intra and extra cellular compartments, reflecting a more severe disease and associated with known poor prognostic markers.

Quilliot et al. [26] previously found a correlation between COVID-19 severity and magnesium levels, where a moderately severe form of the disease was associated with hypomagnesemia, and hypermagnesemia was associated with the existence of nephropathy and a critical severity form. Similarly, Sarvazad et al. [46] found that high magnesium was significantly more prevalent in ICU admitted COVID-19 patients versus outpatients.

These findings support the notion that high magnesium levels serve as a marker for severe COVID-19 disease and renal disfunction, whereas a low magnesium state is an independent risk factor for developing poor disease outcomes.

## 5. Conclusions

Both low and high serum magnesium levels were associated with worse short and long-term survival in a large cohort of hospitalized COVID-19 patients. While low serum magnesium was an independent risk factor for adverse outcomes, high magnesium was correlated with decreased kidney function and markers of severe disease and was not independently associated with worse outcomes after correction for those factors. Thus, low serum magnesium levels upon admission may serve as an available, inexpensive and fast biomarker for poor outcomes and aid in risk stratification of COVID-19 patients. Further prospective studies are needed to establish a potential therapeutic role.

## 6. Limitations

We acknowledge several limitations in our study. First, this was an observational study with a retrospective analysis of collected data. Second, the study was conducted in a single tertiary medical center including patients with de-novo diagnosis of COVID-19 alone, which may contribute to patient selection bias. Thus, the data cannot necessarily be extrapolated to other settings. In addition, as in many studies, we rely on digitally encrypted events, such as coded background diagnoses and discharge diagnoses. Therefore, some data were not available, including data on mechanical ventilation. We nonetheless believe we have gathered sufficient parameters of severe disease that the addition of mechanical ventilation would not alter the conclusions of our work. Lastly, the immediate cause of death was unknown, which precluded making conclusions regarding magnesium levels and mechanism of death.

## Figures and Tables

**Table 1:** Patient characteristics and laboratory parameters by serum magnesium quintiles.

	Q1: ≤1.8 *n* = 326	Q2: 1.9–2.0 *n* = 482	Q3: 2.1–2.1 *n* = 220	Q4: 2.2–2.3 *n* = 271	Q5 ≥ 2.4 *n* = 223	Total *n* = 1522	*p*
Age	69 ± 17	67 ± 17	68 ± 16	66 ± 17	70 ± 16	68 ± 17	0.039
Male gender	166 (50.9)	254 (52.7)	133 (60.5)	181 (66.8)	153 (68.6)	887 (58)	<0.001
BMI	28 ± 6	28 ± 5	28 ± 6	28 ± 6	29 ± 6	28.5 ± 6	0.533
CHF	19 (5.8)	23 (4.8)	11 (5.0)	16 (5.9)	9 (4.0)	78 (5.1)	0.854
IHD	47 (14.4)	65 (13.5)	25 (11.4)	35 (12.9)	27 (12.1)	199 (13.1)	0.854
Hypertension	102 (31.3)	150 (31.1)	69 (31.4)	96 (35.4)	73 (32.7)	490 (32.2)	0.778
Diabetes	78 (23.9)	79 (16.4)	28 (12.7)	32 (11.8)	39 (17.5)	256 (16.8)	<0.001
Dyslipidemia	37 (11.3)	62 (12.9)	20 (9.1)	26 (9.6)	36 (16.1)	181 (11.9)	0.117
Stroke/TIA	39 (12.0)	59 (12.2)	22 (10)	22 (8.1)	24 (10.8)	166 (11.0)	0.460
VTE	13 (4.0)	25 (5.2)	10 (4.5)	13 (4.8)	7 (3.1)	68 (4.5)	0.781
CKD	20 (6.1)	19 (3.9)	11 (5.0)	9 (3.3)	21 (9.4)	80 (5.3)	0.017
COPD	13 (4.0)	21 (4.4)	10 (4.5)	8 (3.0)	10 (4.5)	62 (4.1)	0.878
WBC	7.4 ± 4.5	8 ± 16.8	7.6 ± 4.3	8.3 ± 8.1	9.9 ± 5.3	8.2 ± 10.6	<0.001
Lymphocytes	1.13 ± 2.07	1.9 ± 15.6	1.16 ± 1.27	1.1 ± 1.42	0.95 ± 0.67	1.36 ± 8.88	<0.001
Hemoglobin	12.13 ± 2.18	12.46 ± 2.17	12.8 ± 2	12.6 ± 2	12.6 ± 2	12.5 ± 2	<0.001
Platelets	207 ± 109	207 ± 85	222 ± 103	209 ± 86	246 ± 118	215 ± 100	<0.001
Creatinine	1.24 ± 0.97	1.08 ± 0.86	1.11 ± 0.87	1.2 ± 1.14	1.88 ± 2.02	1.26 ± 1.2	<0.001
CRCL	71.5 ± 31.7	78.2 ± 28.4	77.6 ± 27.6	76.8 ± 80.2	59.5 ± 34.1	73.8 ± 30.9	<0.001
Glucose	150 ± 89	139 ± 67	130 ± 62	139 ± 73	167 ± 86	145 ± 77	<0.001
Sodium	136 ± 6.4	137 ± 5.2	137 ± 4.9	137 ±5.6	139 ± 10	137 ± 6.5	<0.001
Pottasium	4.2 ± 0.68	4.2 ± 0.61	4.45 ± 0.73	4.39 ±0.68	4.48 ± 0.75	4.35 ± 0.69	<0.001
Total calcium	8.7 ± 0.61	8.7 ± 0.58	8.82 ± 0.65	8.69 ± 0.58	8.74 ± 0.67	8.76 ± 0.61	0.053
ALT	28.1 ± 24.8	33.9 ± 33.5	32.7 ± 26	48 ± 127.1	43.2 ± 41.6	36.4 ± 61.3	<0.001
Troponin	149 ± 1057	122 ± 836	183 ± 1534	164 ± 898	96 ± 246	141 ± 975	0.904
CRP	91 ± 86	86 ± 80	93 ± 85	121 ± 91	149 ± 104	104 ± 91	<0.001
LDH	344 ± 160	340 ± 155	399 ± 230	462 ± 481	517 ± 322	397 ± 286	<0.001
Ferritin	544 ± 830	540 ± 698	579 ± 625	844 ±1488	1102 ± 1676	687 ± 1106	<0.001
D-dimer	3074 ± 8001	3160 ± 9094	2042 ± 3964	2861 ± 8578	3682 ± 7913	3004 ± 8027	<0.001
INR	1.14 ± 0.26	1.14 ± 0.35	1.12 ± 0.18	1.18 ± 0.26	1.22 ± 0.48	1.16 ± 0.32	<0.001
Antiplatelet	103 (31.6)	146 (30.3)	67 (30.5)	69 (25.5)	63 (28.3)	448 (29.4)	0.521
Steroids	23 (7.1)	22 (4.6)	5 (2.3)	10 (3.7)	4 (1.8)	64 (4.2)	0.016
Diuretics	65 (19.9)	93 (19.3)	43 (19.5)	52 (19.2)	51 (22.9)	304(20)	0.837
CCB	79 (24.2)	88 (18.3)	39 (17.7)	45 (16.6)	54 (24.2)	305 (20)	0.046
PPI	92 (28.2)	109 (22.6)	52 (23.6)	48 (17.7)	49 (22.0)	350 (23)	0.049

Data are presented as *n* (%) unless otherwise specified. Abbreviations: ALT = Alanine aminotransferase; BMI = body mass index; CCB = calcium channel blockers; CHF = congestive heart failure; CKD = chronic kidney disease; COPD = chronic obstructive pulmonary disorder; CRCL = Creatinine clearance, as calculated by CKD-EPI; CRP = C-reactive protein; IHD = ischemic heart disease; INR = international normalized ratio; LDH = lactate dehydrogenase; PPI = proton pump; TIA = transient ischemic attack inhibitor; VTE = venous thromboembolism; WBC = white blood cell.

**Table 2:** Clinical outcomes according to serum magnesium quintiles.

	Q1: ≤1.8 *n* = 326	Q2: 1.9–2.0 *n* = 482	Q3: 2.1–2.1 *n* = 220	Q4: 2.2–2.3 *n* = 271	Q5 ≥ 2.4 *n* = 223	Total *n* = 1522	*p*
O2 Saturation on admission—% ± SD	93 ± 6.1	93 ± 6.4	92 ± 8	91 ± 7.7	89 ± 10.	92 ± 7.6	<0.001
O2 Saturation ≤93% during hospitalization	266 (84)	367 (80)	169 (80)	222 (85)	200 (92)	1224 (83)	<0.001
Hospitalization length of stay—days ± SD	10.4 ± 17.1	10.6 ± 21.3	12.9 ± 22.8	10.2 ± 16.6	13.4 ± 23.4	11.2 ± 20.3	0.274
Intensive care unit admission	29 (8.9)	39 (8.1)	22 (10)	30 (11.1)	34 (15)	154 (10)	0.007
In hospital mortality	53 (19)	40 (9.5)	21 (10.7)	44 (17.8)	60 (30)	218 (16)	<0.001
Total mortality	115 (35)	113 (23)	59 (26.8)	74 (27.3)	101 (45.3)	462 (30)	<0.001

Data are presented as *n* (%) unless otherwise specified.

**Table 3:** Multivariate analysis model of mortality rate, compared with the 2nd quintile.

	HR	95% CI	*p*
Age	1.050	1.042–1.058	<0.001
Creatinine	1.134	1.072–1.199	<0.001
ALT	0.997	0.995–0.999	0.003
ALK-P	1.001	1.00–1.002	0.051
CRP	1.004	1.003–1.005	<0.001
LDH	1.001	1.001–1.001	<0.001
Desaturation under 93%	0.408	0.261–0.638	<0.001
Sodium	1.033	1.020–1.045	<0.001
Diabetes	1.372	1.087–1.731	0.008
sMg Q2	1		0.009
sMg Q1	1.573	1.204–2.055	<0.001
sMg Q3	1.102	0.793–1.532	0.563
sMg Q4	1.051	0.772–1.431	0.750
sMg sQ5	1.288	0.090–1.727	0.09

Abbreviations: CRP = C-reactive protein; ALT = Alanine aminotransferase; LDH = lactate dehydrogenase.

## Data Availability

The data presented in this study are available on request from the corresponding author. The data are not publicly available due to patient privacy.
